# Cognitive Dysfunction and Mortality After Carotid Endarterectomy

**DOI:** 10.3389/fneur.2020.593719

**Published:** 2021-01-14

**Authors:** Kristiina Relander, Marja Hietanen, Krista Nuotio, Petra Ijäs, Irene Tikkala, Eija Saimanen, Perttu J. Lindsberg, Lauri Soinne

**Affiliations:** ^1^Neuropsychology, Neurocenter, Helsinki University Hospital, Helsinki, Finland; ^2^Clinical Neurosciences, Clinicum, University of Helsinki, Helsinki, Finland; ^3^Neurology, Neurocenter, Helsinki University Hospital, Helsinki, Finland; ^4^Department of Surgery, South Karelia Central Hospital, Lappeenranta, Finland

**Keywords:** carotid endarterectomy, postoperative cognitive dysfunction, mortality, survival, follow-up studies

## Abstract

**Background:** Carotid endarterectomy (CEA) has been associated with both postoperative cognitive dysfunction (POCD) and improvement (POCI). However, the prognostic significance of postoperative cognitive changes related to CEA is largely unknown. The aim of this study was to examine the associations between postoperative cognitive changes after CEA and long-term survival.

**Methods:** We studied 43 patients 1 day before CEA as well as 4 days and 3 months after surgery with an extensive neuropsychological test array, and followed them for up to 14 years. POCD and POCI relative to baseline were determined with the reliable change index derived from 17 healthy controls. Associations between POCD/POCI and mortality within the patient group were studied with Cox regression analyses adjusted for confounders.

**Results:** POCD in any functional domain was evident in 28% of patients 4 days after surgery and in 33% of patients 3 months after surgery. POCI was shown in 23% of patients at 4 days and in 44% of patients at 3 months. POCD at 3 months was associated with higher long-term mortality (hazard ratio 5.0, 95% CI 1.8–13.9, *p* = 0.002) compared with patients with no cognitive decline.

**Conclusions:** Our findings suggest that POCD in a stable phase, 3 months after CEA predicts premature death. Evaluation of postoperative cognitive changes is essential, and POCD in a stable phase after CEA should prompt scrutiny of underlying factors and better adherence to therapies to prevent recurrences and to promote early intervention in imminent deterioration.

## Introduction

Carotid endarterectomy (CEA) is an effective treatment for symptomatic (1, 2) and asymptomatic (3) high-grade carotid stenosis to prevent stroke, but findings on the cognitive consequences of CEA are strikingly mixed. CEA has been associated with both postoperative cognitive dysfunction (POCD), generally found in 10–15% of patients, and postoperative cognitive improvement (POCI) in ~10% patients 1–3 months after the operation (4–10). However, the practical and prognostic significance of postoperative cognitive changes after CEA remain essentially unexplored. POCD related to other types of surgery, such as cardiac (11, 12) and non-cardiac thoracic, abdominal or orthopedic (13) surgery, has been associated with an increased mortality rate. However, studies regarding POCD and survival after revascularization of the respective cerebral hemisphere with CEA are outstandingly scarce. An association between early cognitive dysfunction <24 h after CEA and higher mortality has been shown in patients not using statins at the time of operation but not in statin users (14). However, cognitive functioning in an early postoperative phase can be obscured by surgery-related factors, such as medication, postoperative pain and emotional stress, for which POCD is more reliably defined in a stable phase, preferably more than 30 days after operation (15). It has not yet been established whether efficient cognitive performance as a function of an essentially unbroken neural network of the cerebrum plays a role in later well-being and survival after CEA.

The aim of this study was to examine the associations between postoperative cognitive changes after CEA, assessed in both an acute and a stable postoperative phase, and long-term survival. We hypothesized that the changes in postoperative cognitive performance should have profound prognostic value, especially when examined in the stable phase, 3 months after CEA.

## Materials and Methods

### Setting

This prospective cohort study was carried out within the Helsinki Carotid Endarterectomy Study (HeCES) (6, 16) in the Departments of Neurology and Cardiovascular and Thoracic Surgery at the Helsinki University Hospital, Finland. The study was approved by the ethical committee of the Helsinki University Hospital and performed in accordance with the ethical standards of the Declaration of Helsinki. The participants gave their written informed consent prior to their inclusion in the study.

CEA was performed between February 20, 1997 and March 23, 2000. The patients underwent a comprehensive neuropsychological assessment 1–2 days before surgery and postoperatively 4 days after operation (median 5 days after baseline assessment, range 4–8) and ~3 months after operation (median 95 days after baseline, range 69–164). Post mortem data acquisition was approved by the local Medical Ethics Committee, the National Institute for Health and Welfare and Statistics Finland. Follow-up medical history was collected from hospital archives and electronic patient records beginning from CEA until April 30, 2012 or death. Causes of death were collected from death certificates archived at Statistics Finland.

The control group participated in neuropsychological testing but was not included in long-term follow-up. They underwent neuropsychological assessments three times at similar time intervals as the patients (second assessment median 5 days after baseline, range 4–7; third assessment median 117 days after baseline, range 89–180).

### Participants

A cohort of 44 consecutive CEA patients of the HeCES study (6, 16) were enrolled in this substudy upon patient consent and availability of imaging facilities and the necessary personnel during the time frames as defined by the protocol. The participants were independent in activities of daily living, had no potential cardiogenic origin of emboli, no history of previous ipsilateral CEA or radiotherapy, and had a surgically accessible symptomatic (TIA or minor stroke, i.e., no need for inpatient rehabilitation or a deficit that would be expected to be a limiting factor in the neuropsychological examination) or asymptomatic unilateral carotid stenosis of at least 70% in digital subtraction angiography (NASCET criteria).

The level of education was scored on a three-code scale according to the Finnish education system: basic level (compulsory education requiring 6–9 years of education), middle level (vocational training, matriculation examination and/or bachelor's degree requiring 8–15 years of education), or higher level (university level master's degree or higher requiring a minimum of 16 years of education). Occupational attainment was scored using a simple three-code scale: physical or manual labor workers, skilled manual professionals, and non-manual white collar workers.

A control population was included in order to account for normal variation and practice effects in neuropsychological tests in order to define criteria for POCD/POCI. The control population consisted of 17 volunteers matched for age, gender, and education and occupation level of the patients. The control population had no medications, no signs or history of cardiovascular or neurological morbidity, no excessive alcohol intake over longer periods and no family history of neurodegenerative diseases.

### Surgical Management and Postoperative Care

Patients were operated under general anesthesia with routine hemodynamic and transcranial Doppler monitoring by the same surgeon with a standard approach. Sedation, induction, muscle relaxation, and maintenance of anesthesia were carried out according to the hospital practice. Shunting was performed in three patients on the basis of stump pressure. A 1.5 Tesla magnetic resonance (MR) imaging of the brain was obtained 1 day before surgery and ~4 days and 3 to 4 months after surgery.

At discharge, all patients were deemed to have adequate preventive medication, and according to the Finnish healthcare system, they were referred to primary healthcare for the continued management and follow-up of vascular risk factors.

### Neuropsychological Assessment

The test battery was selected from established neuropsychological tests and classified into seven functional domains. *Learning* included the Logical memory subtest of the Rivermead Behavioral Memory test, the Auditory verbal learning test (10 words, sum of trials 1 to 5) and the Rey visual learning test (15 drawings, sum of trials 1 to 5). *Delayed memory* included delayed recall of Logical memory, the Auditory verbal learning test and the Rey visual learning test as well as recognition of the Rey visual learning test. *Working memory* comprised Digit span forwards and backwards. *Executive functioning* included the Letter Cancellation test (time to complete), the Trail Making test, part B subtracted by part A (times to complete), the Stroop test, the Word subtest subtracted by the Color subtest (times to complete) and Verbal phonemic fluency. *Motor dexterity* comprised Finger tapping and the Purdue Pegboard (hand contralateral to stenosis in both tests). *Processing speed* included the Trail Making test, part A (time to complete) and the Stroop Color subtest (time to complete). *Reasoning* included the Similarities and Block Design subtests of the Wechsler Adult Intelligence Scale—revised (reasoning was not assessed at 4 days). In order to minimize learning effects, parallel memory test versions were used in repeated measurements.

Missing values were not imputed. All test scores were standardized using healthy controls' baseline performance as reference. Timed z-scores were inverted so that negative z-scores always indicated worsening of cognitive performance. Domain-wise scores were formed by averaging the standardized z-scores of each patient within each functional domain.

### Risk Factor Assessment

Smoking was measured in pack years (smoking years^*^amount of smoked packs of cigarettes per day). Preoperative depressive symptoms were assessed with the short 13-item version of the Beck Depression Inventory (BDI) (17). Symptomatic stenosis (TIA or minor stroke), body mass index (BMI) as well as occurrence of preoperative stroke, high blood pressure, diabetes and dyslipidemia were recorded at baseline. Statin use was recorded both preoperatively (>4 weeks usage before surgery) and at follow-up. Degree of preoperative white matter hyperintensities (HI) was assessed on a four-code scale (HI-1, small and focal, <5 mm; HI-2, larger focal, 6–10 mm; HI-3, focal confluent, 11–25 mm; HI-4, diffusely confluent) and categorized into HI-1 or more.

### Statistical Methods

Statistical analyses were performed with IBM SPSS 25. *P*-values below 0.05 were considered significant. Pre-existing cognitive impairment was defined as below two standard deviations of performance relative to the control group's baseline. Presence of POCD/POCI at 4 days and 3 months was determined within each domain using the reliable change index derived from 17 control participants. This approach allows for comparison between preoperative and postoperative performance within individual patients while taking normal variation and practice effects of the control group into account (18–20). The criterion of domain-wise POCD/POCI was set to domain-specific z-score ± 2 and a patient with POCD/POCI in any domain was classified as having POCD/POCI. Univariate groupwise comparisons between patients and controls, between symptomatic and asymptomatic patients and between patients that did or did not show POCD were made with independent samples *t*-tests or Mann–Whitney *U* tests for continuous variables and with χ^2^ tests or Fisher's exact tests for categorical variables. Cox regression analyses were performed in three steps within the patient group. First, associations between separate patient characteristics and mortality were examined with Cox regression analyses adjusting for gender and age at surgery. Second, patient characteristics with *p* < 0.2 were entered into the model simultaneously. Also, the interaction between significant patient characteristics was checked at this phase. Finally, cardiovascular risk factors (symptomatic stenosis, smoking, BMI, high blood pressure, diabetes, and dyslipidemia) were added into the model. Kaplan–Meier curves were formed according to significant patient characteristics and compared with the log-rank test.

## Results

### Participants

After a follow-up time of 12 to 14 years after the operation, one patient declined to further participate in the study. The remaining 43 patients (21 symptomatic, 22 asymptomatic) were included in this study. Out of the final population, 16 patients were deceased, and 27 surviving patients agreed to participate and gave written informed consent. Median time until end of follow-up or death was 12.5 years (range 1.8 to 14.6).

Characteristics of the patient population are shown in Table 1 and have previously been described in detail (6). There were no significant differences in age, gender, education or occupation level between symptomatic and asymptomatic patients or between controls and patients (Table 1).

**Table 1:** Baseline characteristics of the study population.

	**Symptomatic patients (*N* = 21)**	**Asymptomatic patients (*N* = 22)**	***P*-value**	**All patients (*N* = 43)**	**Controls (*N* = 17)**	***P*-value**
Age, years	62.7 ± 9.3	65.1 ± 8.4	0.38	63.9 ± 8.8	63.6 ± 6.8	0.89
Gender, male	15 (71%)	13 (59%)	0.40	28 (65%)	12 (71%)	0.69
Education years	8 (5)	10 (4)	0.34	8 (3)	9 (6)	0.26
Education class			0.56			0.73
Basic level	12 (57%)	10 (46%)		22 (51%)	7 (41%)	
Middle level	6 (29%)	10 (46%)		16 (37%)	7 (41%)	
Higher level	3 (14%)	2 (9%)		5 (12%)	3 (18%)	
Occupation class			0.61			1.00
Manual labor	4 (19%)	7 (32%)		11 (26%)	4 (24%)	
Skilled manual	11 (52%)	9 (41%)		20 (47%)	8 (47%)	
Non-manual	6 (29%)	6 (27%)		12 (28%)	5 (29%)	

*Data are presented as mean ± SD, median (interquartile range) or N (%)*.

*P-values are from t-tests, Mann–Whitney U tests, χ^2^ tests and Fisher's exact tests*.

### Cognitive Performance

There were missing values in 21 out of 3,300 single values of the neuropsychological data matrix. As a result of domain-averaging, there was only one missing value in POCD and POCI values (reasoning at 3 months).

Domain-wise cognitive performance of patients and controls is shown in Table 2. Pre-existing cognitive impairment was detected in 2 (5%) patients in learning, no patients in delayed memory, 1 (2%) in working memory, 17 (40%) in executive functioning, 6 (14%) in speed, 4 (9%) in motor dexterity and 9 (21%) in reasoning. None of the controls exhibited pre-existing cognitive impairment in any domain.

**Table 2:** Cognitive performance of patients and controls in each measurement.

	**Patients**			**Controls**		
**Cognitive domain**	**Baseline**	**4 days**	**3 months**	**Baseline**	**4 days**	**3 months**
Learning	−0.52 ± 0.97	−0.50 ± 1.00	−0.17 ± 0.96	0 ± 0.81	0.19 ± 0.86	0.47 ± 0.58
Delayed memory	−0.49 ± 0.85	−0.43 ± 0.97	−0.19 ± 0.86	0 ± 0.77	0.21 ± 0.85	0.40 ± 0.68
Working memory	−0.43 ± 0.78	−0.57 ± 1.17	−0.35 ± 1.02	0 ± 0.92	0.17 ± 0.92	0.42 ± 1.15
Executive functioning	−1.51 ± 1.56	−1.24 ± 1.64	−0.92 ± 1.49	0 ± 0.54	0.03 ± 0.83	0.05 ± 0.77
Speed	−0.36 ± 1.21	−0.33 ± 1.46	−0.44 ± 1.53	0 ± 0.92	0.55 ± 0.60	0.57 ± 0.57
Motor dexterity	−0.66 ± 0.89	−0.79 ± 1.03	−0.61 ± 1.20	0 ± 0.68	0.29 ± 0.58	0.33 ± 0.74
Reasoning	−0.79 ± 1.31		−0.62 ± 1.17	0 ± 0.87		0.24 ± 0.96

*Results are expressed as mean z-scores ± SD standardized relative to the baseline of controls*.

*Reasoning was not assessed at 4 days*.

No clinical strokes were detected after CEA, but postoperative MR imaging showed a new minor asymptomatic watershed stroke and microhemorrhages in one patient who did not show POCD or POCI in either measurement. POCD in any domain was evident in 12 (28%) patients at 4 days and 14 (33%) at 3 months. POCI, in turn, was found in 10 (23%) patients at 4 days and 19 (44%) at 3 months. POCD and POCI were generally evident in single domains: in both assessments, only four patients showed POCD and one patient POCI in more than one domain. Domain-specific POCD occurrences are shown in Table 3.

**Table 3:** Cognitive changes at 4 days and 3 months.

	**4 days**	**3 months**
POCD in any domain	12 (28%)	14 (33%)
Learning	3 (7%)	5 (12%)
Delayed memory	1 (2%)	3 (7%)
Working memory	2 (5%)	2 (5%)
Executive functioning	5 (12%)	3 (7%)
Speed	2 (5%)	5 (12%)
Motor dexterity	5 (12%)	6 (14%)
Reasoning		0 (0%)
POCI in any domain	10 (23%)	19 (44%)
Learning	1 (2%)	1 (2%)
Delayed memory	2 (5%)	0 (0%)
Working memory	0 (0%)	1 (2%)
Executive functioning	7 (16%)	13 (30%)
Speed	1 (2%)	1 (2%)
Motor dexterity	0 (0%)	2 (5%)
Reasoning		2 (5%)

*Data are presented as N (%)*.

*POCD, postoperative cognitive dysfunction; POCI, postoperative cognitive improvement*.

*Both determined with the reliable change index*.

*Reasoning was not assessed at 4 days*.

Associations between POCD and patient characteristics are shown in Table 4. In sum, POCD at 4 days was not associated with demographic variables, risk factors, white matter hyperintensities, symptomatic stenosis or stroke, whereas POCD at 3 months was associated with less frequent continuous (>4 weeks) use of statins before CEA but no other confounders.

**Table 4:** Differences in baseline characteristics between patients with or without postoperative cognitive dysfunction.

	**4 days**			**3 months**		
	**POCD (*N* = 12)**	**No POCD (*N* = 31)**	***P*-value**	**POCD (*N* = 14)**	**No POCD (*N* = 29)**	***P*-value**
Age, years	69.5 (20)	62 (15)	0.25	62.5 (16)	62 (18)	0.50
Gender, male	7 (58%)	21 (68%)	0.72	4 (29%)	11 (38%)	0.74
Education, years	8 (2.8)	8 (4)	0.30	8.5 (4.5)	8 (4)	0.48
Education class			0.31			0.65
Basic level	8 (67%)	14 (45%)		8 (57%)	14 (48%)	
Middle level	4 (33%)	12 (39%)		4 (29%)	12 (41%)	
Higher level	0 (0%)	5 (16%)		2 (14%)	3 (10%)	
Occupation class			0.62			0.24
Manual labor	4 (33%)	7 (23%)		5 (36%)	6 (21%)	
Skilled manual	6 (50%)	14 (45%)		4 (29%)	16 (55%)	
Non-manual	2 (17%)	10 (32%)		5 (36%)	7 (24%)	
Smoking, pack years	36 (17.5)	20 (30)	0.07	25 (35.6)	29 (30)	0.91
Body mass index	27.2 (5.8)	27.3 (4.6)	0.51	27.0 (5.9)	27.3 (4.5)	0.45
Dyslipidemia	8 (67%)	17 (55%)	0.48	9 (64%)	16 (55%)	0.57
Statin use (>4 weeks)	3 (25%)	10 (32%)	0.73	1 (7%)	12 (41%)	0.03*
High blood pressure	6 (50%)	17 (55%)	0.78	7 (50%)	16 (55%)	0.75
Diabetes	2 (17%)	6 (19%)	1.00	3 (21%)	5 (17%)	1.00
Depressive symptoms	8 (7)	3.5 (5)	0.06	3.5 (3)	4.5 (7)	0.27
Stroke	4 (33%)	11 (36%)	1.00	5 (36%)	10 (35%)	1.00
White matter hyperintensities			0.26			0.07
HI-1	5 (42%)	19 (61%)		5 (36%)	19 (66%)	
HI-2	6 (50%)	8 (26%)		6 (43%)	8 (28%)	
HI-3	0 (0%)	4 (13%)		2 (14%)	2 (7%)	
HI-4	1 (8%)	0 (0%)		1 (7%)	0 (0%)	
Symptomatic carotid stenosis	8 (67%)	13 (42%)	0.15	6 (43%)	15 (52%)	0.59

*Data are presented as median (interquartile range) or N (%)*.

*White matter hyperintensities were categorized into HI-1 or more in statistical analysis*.

P-values (*p < 0.05) are from Mann–Whitney U tests, χ^2^ tests or Fisher's exact tests. Statistically significant p-values are bolded.

### Mortality

Causes of death were ischemic heart disease (seven patients), Alzheimer's disease (two patients), malignancy (two patients) or miscellaneous (five patients). Both patients who died of Alzheimer's disease had POCD in both postoperative assessments.

**Figure 1:**
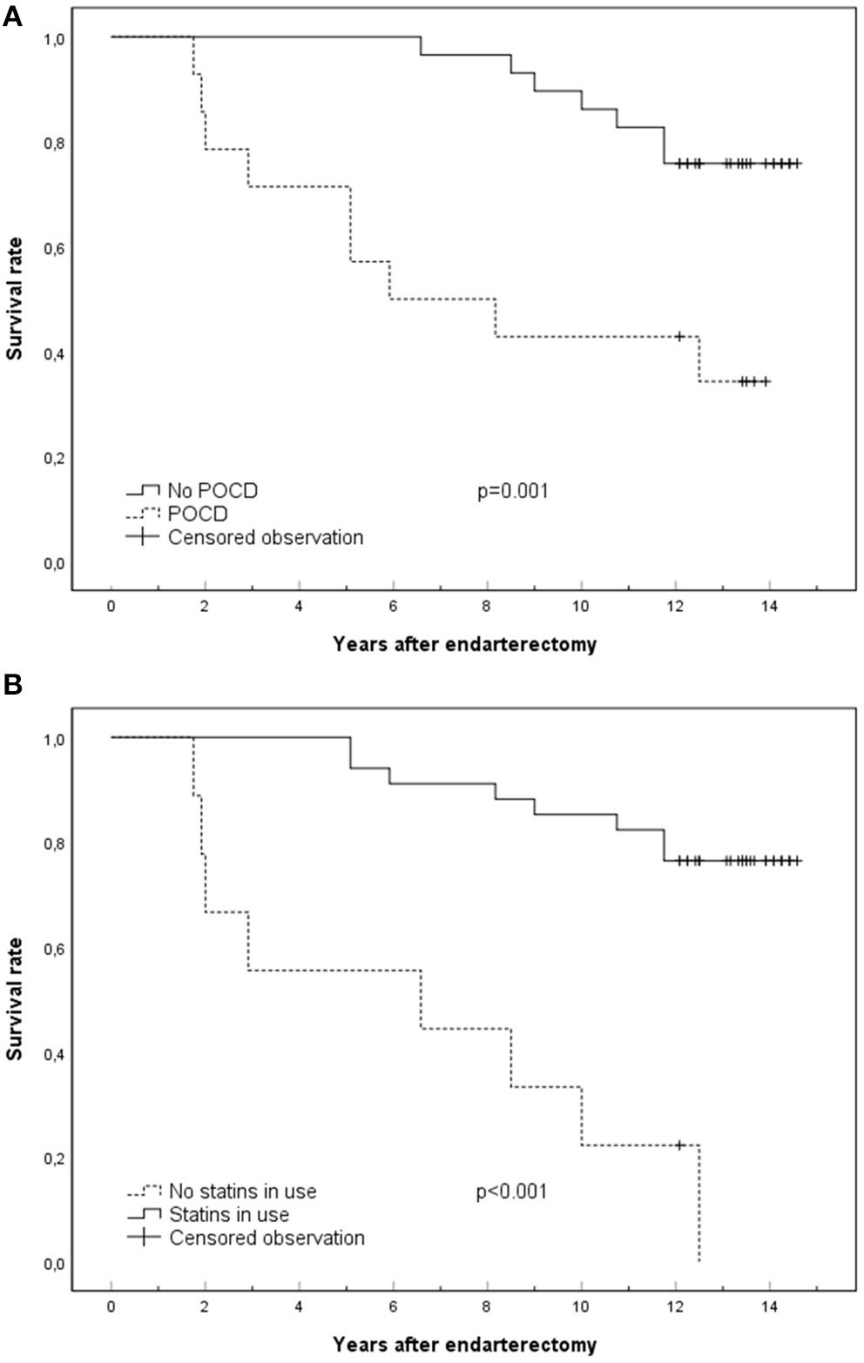
Kaplan–Meier curves presenting major factors that affect survival: POCD at 3 months **(A)** and statin use at the time of death or end of follow-up **(B)**. Censored observations are surviving patients at the end of follow-up. POCD, postoperative cognitive dysfunction.

Results of survival analyses adjusted for gender and age at surgery are shown in Table 5 and Figure 1. Major factors affecting survival were POCD at 3 months (HR 5.0, 95% CI 1.8–13.9, *p* = 0.002) and not using statins at follow-up (HR 10.7, 95% CI 3.1–37.7, *p* < 0.001). Both factors remained significant in the multivariate analyses even when symptom status of stenosis and cardiovascular risk factors were added to the models. The interaction between POCD at 3 months and statins at follow-up was not significant (*p* = 0.93).

**Table 5:** Cox regression analyses adjusted for age and gender.

	**First analyses**			**Second analysis**			**Third analysis**		
	**HR**	**95% CI**	***P*-value**	**HR**	**95% CI**	***P*-value**	**HR**	**95% CI**	***P*-value**
Education, years	1.0	0.9–1.2	0.70						
Education class			0.59						
Basic level	1.0								
Middle level	1.3	0.4–4.0	0.67						
Higher level	0.5	0.1–2.5	0.39						
Occupation class			0.23			0.46			0.26
Manual labor	1.0			1.0			1.0		
Skilled manual	0.4	0.1–1.2	0.10	0.6	0.2–2.2	0.45	0.5	0.1–3.3	0.47
Non-manual	0.5	0.1–1.6	0.2	0.4	0.1–1.7	0.22	0.2	0.0–1.5	0.11
Smoking, pack years	1.0	1.0–1.0	0.93				1.0	0.9–1.0	0.45
Body mass index	1.0	0.9–1.1	0.59				1.1	0.9–1.3	0.33
Dyslipidemia	0.7	0.2–1.8	0.43				2.2	0.5–10.5	0.31
High blood pressure	1.5	0.5–4.1	0.43				6.4	0.9–45.1	0.06
Diabetes	0.6	0.1–2.8	0.53				0.1	0.0–0.9	0.04
Depressive symptoms	1.0	0.9–1.2	0.93						
Stroke	1.4	0.5–3.9	0.50						
White matter hyperintensities	0.4	0.1–2.1	0.30						
Symptomatic carotid stenosis	0.9	0.3–2.5	0.86				0.6	0.2–2.4	0.48
No statins (<4 weeks) at baseline	2.0	0.6–6.5	0.23						
No statins at follow-up	10.7	3.1–37.7	<0.001^***^	7.9	2.0–31.5	0.003^**^	8.6	1.6–46.3	0.012^*^
POCD at 4 days	1.6	0.6–4.5	0.36						
POCD at 3 months	5.0	1.8–13.9	0.002^**^	3.8	1.3–11.0	0.016^*^	16.3	2.4–108.7	0.004^**^
POCI at 4 days	1.5	0.5–4.4	0.42						
POCI at 3 months	0.8	0.3–2.4	0.73						

*In first analysis, patient characteristics were entered separately*.

*In second analysis, patient characteristics with p < 0.2 were entered simultaneously*.

*In third analysis, symptom status of stenosis and cardiovascular risk factors were added into the multivariate model*.

*HR, hazard ratio; CI, confidence interval*.

* *p < 0.05*,

** *p < 0.01*,

*** *p < 0.001. Statistically significant p-values are bolded*.

## Discussion

We found that POCD after CEA is associated with a 5-fold increase in mortality after a follow-up time of 12 to 14 years. Furthermore, this association seems even stronger when other potential confounders, such as cardiovascular risk factors and symptom status of carotid stenosis, are taken into account. The association between POCD and mortality was only evident when POCD was evaluated in a stable phase, 3 months after CEA. In contrast, POCD shown in an acute phase, 4 days post-surgery, was not associated with survival. Furthermore, POCI in either assessment had no prognostic value on mortality. Our findings emphasize the significance of recognizing chronic POCD that may predict significantly poorer long-term outcomes.

Our results side with earlier findings on the association between mortality and stable phase POCD after non-cardiac (13) and cardiac (11, 12) surgery, and further expand these findings to the revascularization of cerebral circulation with CEA. Our study also amplifies the research of Heyer et al. (14) who found a relationship between early (<24 h) cognitive decline after CEA and mortality in patients not using statins at the time of surgery (14). With a different study design, we were able to show that the prognostic value of POCD is much larger [hazard ratio 5.0 compared to 1.6 in Heyer et al. (14)] and evident in the whole patient group regardless of statin use when POCD is defined in a stable phase, 3 months after CEA. In fact, POCD assessed 4 days after surgery was not significantly related to long-term survival in the present study. These findings are in accordance with the view that POCD is more reliably defined after 30 days post-surgery, when confounding factors such as pain, medications, and postoperative emotional stress are stabilized (15).

In contrast to postoperative deterioration, no significant associations were found between mortality and POCI in either postoperative assessment. These findings are in agreement with our recent findings revealing that POCD, but not POCI, was associated with the course of long-term cognitive performance after cardiac surgery (21). However, POCI after cardiac surgery is probably related to better cardiac functioning that improves overall health, (22) while after CEA it may follow from improvement in cerebral hemodynamics and the associated neuronal metabolism (7, 8, 10). The most often improved cognitive domain in the present study was executive functioning, a complex set of cognitive functions requiring activation in widespread neural circuits, particularly in the anterior parts of the brain (23) that are, indeed, provided with a blood supply from the carotid arteries. However, improvement of these functions probably facilitates activities demanding complex cognitive abilities rather than overall health or health-related behavior. Thus, the long-term effects of POCI are likely to improve daily functioning and working ability rather than survival rate *per se*.

There are many possible explanations for the associations between POCD and mortality after CEA. In general, the beneficial long-term effects of CEA have been established: the risk for major stroke or death is lower in symptomatic carotid patients undergoing CEA compared with non-surgical controls, (1, 2) and CEA also reduces the risk for stroke in asymptomatic carotid patients (3). However, our findings imply that POCD after CEA indicates a risk for poor long-term prognosis. POCD may reflect more severe perioperative neural injury (13, 22) and lead to vulnerability for cumulating cardiovascular or neurovascular injury. POCD related to perioperative neural injury would most probably be seen in cognitive functions of the anterior parts of the brain supplied with blood flow by the carotid arteries, in particular executive functions. However, in the present study, POCD was evident rather equally in several cognitive domains. While our sample size does not allow for firm conclusions, our results may imply that POCD seen in several cognitive domains is related to reasons more varied than simply perioperative injury. Furthermore, many causes of death in the present study were not related to cardiovascular injury, suggesting a more general association between POCD and poor long-term outcome. POCD may reflect predisposing patient-related factors, such as age and education level (22, 24), which did not, however, reach significance in the present study. It may also act as an overall marker for poorer overall health and vulnerability for various medical problems (25). Moreover, persistent POCD may limit the patient's ability to engage in a healthy lifestyle, as well as seek and commit to medical care (13), leading to suboptimal secondary prevention. Of particular interest, POCD may in some patients reflect undiagnosed, early-phase neurodegenerative disease, as suggested by associations between POCD after orthopedic surgery and biomarkers of Alzheimer's disease (26, 27). Indeed, although not diagnosed at the time of recruitment, a few patients in the POCD group, but none in the no POCD group, later died of Alzheimer's disease. Associations between POCD after CEA and neurodegenerative disease have not yet been studied, but associations between POCD after cardiac surgery and long-term cognitive decline have been found (11, 21, 28, 29). Future studies should address the associations between POCD after CEA, long-term cognitive deterioration and neurodegenerative diseases. In sum, we suggest that POCD after CEA may relate to various pre-, peri- and postsurgical medical problems as well as poor health choices and adherence to treatment, and should be taken as a general indicator of health vulnerability.

In accord with previous results (30), POCD at 3 months was related to less frequent continuous (>4 weeks) use of statins at baseline, while no other baseline characteristics were predictive of POCD. This finding may reflect vulnerability to POCD in patients with low compliance and suboptimal treatment of cardiovascular disease. Furthermore, no statin use at follow-up was predictive of worse survival in the present study. This finding is in agreement with results in another report of the same study cohort by our group with a larger sample, showing a dose-related effect of statin use on mortality (16). However, in contrast to earlier results (14), statin use at baseline was not predictive of survival in the present study, and we found no interaction between POCD, mortality and statin use. Deviant results could be explained by differences in study design. First, Heyer et al. (14) based their findings on early cognitive decline (<24 h post-surgically), a time point potentially biased by surgery-related confounding factors (15). Second, they defined statin use at baseline and did not have access to later records of statin use (14). However, the lack of significant interaction between POCD, mortality and statin use in the present study may also result from reduced statistical power due to our small sample size.

The strengths of this study include an extensive neuropsychological evaluation, specific criteria for POCD based on a comparable control population, consideration of all major cardiovascular risk factors and a long follow-up time with a very small (2%) dropout rate. The most important limitation is the restricted sample size that may reduce the power to recognize potential factors that may be associated with POCD or modulate the connections between POCD and mortality. Also, the limited sample size did not allow for investigation of the associations between POCD and mortality separately within the carefully designed cognitive domains in the present study. However, our domain-specific approach allowed for a more reliable definition of POCD than compound scores or individual tests. Notably, the rather robust association between POCD and mortality was clearly evident with the present limited cohort, while none of the conventionally recognized preoperative risk factors could reliably predict survival. This finding highlights the importance of POCD as a converging risk factor that should not be neglected at postoperative evaluations, as well as the need for further inquiry to corroborate and characterize the association in detail.

In conclusion, our study suggests that patients with evident decline in a stable phase after carotid surgery are at risk for poor long-term survival. Our findings underscore the importance of recognizing postoperative cognitive decline after carotid endarterectomy and taking it as an indication for intensified medical surveillance and care, including investigation of underlying factors and promoting compliance to treatment. In order to further improve clinical practice, future larger-scale investigation is needed to determine the cognitive domains that are most vulnerable to POCD and most sensitive to predicting survival after CEA, and whether the outcome may be improved by intervention measures.

## Data Availability Statement

The datasets presented in this article are not readily available because requests for sharing of data will be given individual consideration after additional approval for sharing by the local Ethics committee. Requests to access the datasets should be directed to Kristiina Relander, kristiina.relander@helsinki.

## Ethics Statement

The studies involving human participants were reviewed and approved by the ethical committee of the Helsinki University Hospital. The patients/participants provided their written informed consent to participate in this study.

## Author Contributions

PL, MH, KN, KR, PI, LS, and ES contributed to the conception and design of the study. The data acquisition was designed and put into practice by IT, LS, KN, and PI. The data analysis was performed by KR who also drafted the article manuscript, which was critically revised by PL, MH, and LS. All authors discussed the analyses, commented on the manuscript, and approved the final submitted version of the manuscript.

## Conflict of Interest

The authors declare that the research was conducted in the absence of any commercial or financial relationships that could be construed as a potential conflict of interest.
